# Transcriptomics reveals immune-metabolism disorder in acute-on-chronic liver failure in rats

**DOI:** 10.26508/lsa.202101189

**Published:** 2021-12-01

**Authors:** Hozeifa M Hassan, Qun Cai, Xi Liang, Jiaojiao Xin, Keke Ren, Jing Jiang, Dongyan Shi, Yingyan Lu, Tan Li, Yuxin Shang, Lulu He, Xi Chen, Suwan Sun, Peng Li, Beibei Guo, Jiaxian Chen, Hui Yang, Wen Hu, Xin Chen, Jun Li

**Affiliations:** 1 State Key Laboratory for Diagnosis and Treatment of Infectious Diseases, National Clinical Research Center for Infectious Diseases, Collaborative Innovation Center for Diagnosis and Treatment of Infectious Diseases, The First Affiliated Hospital, Zhejiang University School of Medicine, Hangzhou, China; 2 Precision Medicine Center, Taizhou Central Hospital (Taizhou University Hospital), Taizhou, China; 3 Key Laboratory of Cancer Prevention and Therapy Combining Traditional Chinese and Western Medicine, Tongde Hospital of Zhejiang Province, Hangzhou, China; 4 Imperial College London, South Kensington Campus, London, UK; 5 Shanghai Pinghe School, Shanghai, China; 6 Institute of Pharmaceutical Biotechnology and The First Affiliated Hospital Department of Radiation Oncology, Zhejiang University School of Medicine, Hangzhou, China; 7 Joint Institute for Genetics and Genome Medicine Between Zhejiang University and University of Toronto, Zhejiang University, Hangzhou, China

## Abstract

Acute-on-chronic liver failure (ACLF) is clinical syndrome with high mortality rate. This study aimed to perform detailed transcriptomic analysis in liver cirrhosis–based ACLF rats to elucidate ACLF pathogenesis. ACLF was induced by combined porcine serum with D-galactosamine and lipopolysaccharide. Gene expression profile of liver tissues from ACLF rats was generated by transcriptome sequencing to reveal the molecular mechanism. ACLF rats successfully developed with typical characteristics. Total of 2,354/3,576 differentially expressed genes were identified when ACLF was compared to liver cirrhosis and normal control, separately. The functional synergy analysis revealed prominent immune dysregulation at ACLF stage, whereas metabolic disruption was significantly down-regulated. Relative proportions of innate immune–related cells showed significant elevation of monocytes and macrophages, whereas adaptive immune–related cells were reduced. The seven differentially expressed genes underlying the ACLF molecular mechanisms were externally validated, among them *THBS1*, *IL-10*, and *NR4A3* expressions were confirmed in rats, patient transcriptomics, and liver biopsies, verifying their potential value in the ACLF pathogenesis. This study indicates immune-metabolism disorder in ACLF rats, which may provide clinicians new targets for improving intervention strategies.

## Abstract (precis)

Liver tissue transcriptomics of liver cirrhosis (LC)–based acute-on-chronic liver failure (ACLF) rats reveal immune-metabolism disorder as the core mechanism underlying ACLF development and prognosis.

## Introduction

Acute-on-chronic liver failure (ACLF) is a clinical syndrome that develops in patients with chronic liver diseases (CLDs) after a precipitating event and associated with a high mortality rate because of systemic multiorgan failure (Wu et al, 2018; Arroyo et al, 2020). The pathogenesis, biomarkers, and management of ACLF have become points of interest, as specific treatment for ACLF is still lacking, whereas organ support and complication prevention are common alternatives; if medical treatment fails, transplantation is the only option for eligible patients (Mochida et al, 2018; Moreau et al, 2020). ACLF precipitants are considered the driving force triggering the immune response and inflammatory stress that ultimately initiates ACLF in patients with underlying CLDs or cirrhosis (Hernaez et al, 2017; Zaccherini et al, 2020). Clinically, the two major prospective multicenter reports of ACLF to date, the chronic liver failure (CLIF) Consortium Acute-On-Chronic Liver Failure in Cirrhosis (CANONIC) study, indicated that an intense systemic inflammatory response is the main cause of acute deterioration in patients with alcoholic liver disease-related and hepatitis C virus–related ACLF (Clària et al, 2016). The main features of the systemic inflammation are presence of pathogen-associated molecular patterns of bacterial origin and damage-associated molecular patterns released by dying cells, which activates pattern-recognition receptors of the innate immune system that ultimately causes organ failures in ACLF (Arroyo et al, 2021; Zaccherini et al, 2021). In the meantime, our recent large prospective multicenter study (Chinese Group on the Study of Severe Hepatitis B, COSSH) documented that hepatitis B virus (HBV)–related ACLF (HBV-ACLF) is characterized by an excessive immune response triggered by HBV exacerbation that drives chronic hepatitis B or liver cirrhosis (LC) to acute-on-chronic hepatic dysfunction and ACLF. This dysregulation of immune responses leads to metabolic disorder and inflammation, which induces multiorgan failure in HBV-ACLF patients (Li et al, 2021).

Despite the availability of animal models representing liver diseases such as acute liver failure, CLDs, and fulminant hepatic failure (Shi et al, 2017; Nevzorova et al, 2020), ACLF remains a severe clinical entity, necessitating the establishment of appropriate and reliable animal model, as the availability of such animal model is crucial to fully understand ACLF disease pathogenesis. The key elements in establishing such a model are to initiate liver fibrosis or its advanced form cirrhosis, which represents the common final pathway of most types of CLDs (Engelmann et al, 2020), followed by acute insult to ultimately develop ACLF. At present, available animal models that accurately mimic ACLF are rare, and the currently available models are inconsistent with ACLF pathophysiology.

Transcriptome analysis plays an important role in depicting the molecular fingerprints of disease pathology and prognosis by pinpointing potential biomarkers and intervention pathways (Jiang et al, 2015), which helps provide insight into the mechanisms underlying ACLF development. As the key problem hindering the study of ACLF liver transcriptomics is the difficulty of accessing human liver samples (MacParland et al, 2018), our previous study based on PBMCs transcriptomics indicated immune-metabolism disorder is a core axis of disease development and progression in patients with HBV-ACLF (Li et al, 2021). Hence, in the present study, we aimed to perform transcriptomics-based dataset in LC-based ACLF rat model that would replicates the pathological process of ACLF in humans. The gene expression profile of ACLF liver tissues collected at different disease stages was generated by transcriptome sequencing to provide in-depth insights to reveal the biological pathways and molecular mechanisms that underlie ACLF development and progression.

## Results

### ACLF establishment on the basis of preexisting LC

**Figure 1.**
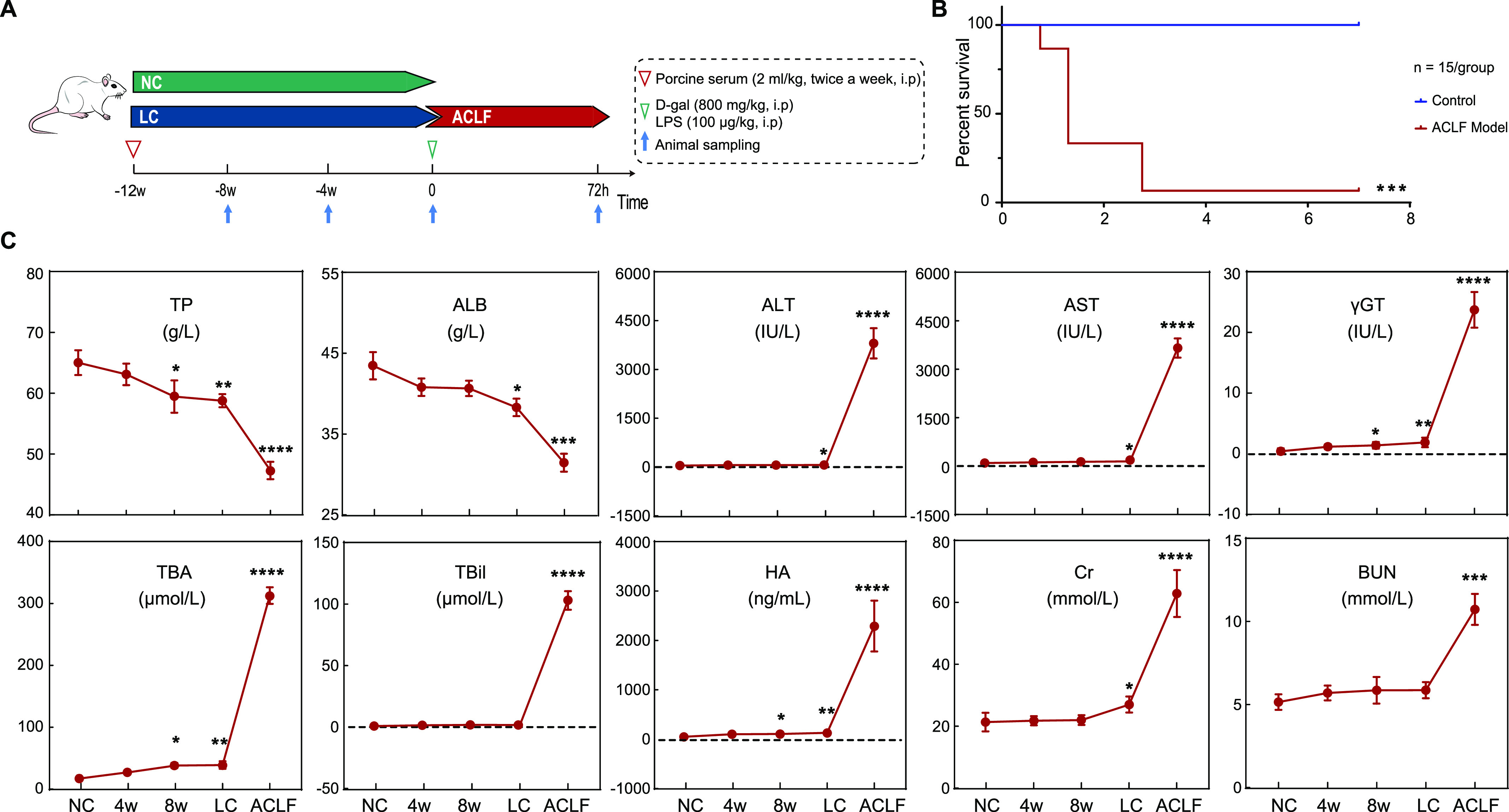
To establish ACLF, SD rats were intraperitoneally co-administered porcine serum twice a week for 12 wk with D-gal/LPS acute insult. **(A)** Schematic representation highlighting ACLF rat model development. **(B)** Kaplan–Meier survival analysis of rats during the ACLF period. The survival of ACLF and normal SD rats was observed for 1 wk (n = 15/group). **(C)** Influence of the serum biomarker profile. Data are presented as the mean ± SD, n = 8–16 for each bar. *P < 0.05, **P < 0.01, ***P < 0.001, ****P < 0.0001 versus NC. ACLF, acute-on-chronic liver failure; ALB, albumin; ALT, alanine transaminase; AST, aspartate transaminase; BUN, blood urea nitrogen; Cr, serum creatinine; HA, hyaluronic acid; LC, liver cirrhosis; NC, normal control; TBA, total bile acids; TBil, total bilirubin; TP, total protein; γGT, gamma-glutamyl transferase.

Fig 1A illustrated the schematic diagram of ACLF rat model establishment. To develop ACLF, rats with stable cirrhosis generated after 12 consecutive weeks of porcine serum (PS) administration were further intraperitoneally administered D-galactosamine (D-gal) and LPS co-treatment to induce an acute state of liver failure and the mortality rate of ACLF rats within 3 d was 93.3% (Fig 1B). As shown in Fig 1C, PS-induced LC development was observed in eight randomly selected rats at 4, 8, and 12 wk after PS injection, in which blood biomarkers showed significant time-dependent alterations, whereas typical LC was generated in rats following 12 wk (herein will be labelled as the LC group). ACLF rats showed significant reductions of total protein (TP) and albumin (ALB) (*P* < 0.001) compared to values obtained from both the normal control (NC) and LC groups. ACLF generation was associated with significant elevation of alanine transaminase (ALT), aspartate transaminase (AST), gamma-glutamyl transferase (γGT) total bile acids (TBAs), and total bilirubin (TBil) (*P* < 0.0001). Serum hyaluronic acid (HA) measurements revealed significant increases in the cirrhotic rats (*P* < 0.01) and continued elevation, reaching peak levels (*P* < 0.0001) in the ACLF state. Furthermore, in comparison to those in the NC and LC groups, serum creatinine (Cr), and blood urea nitrogen (BUN) levels were considerably elevated in the ACLF group, reflecting the possible incidence of renal dysfunction.

**Figure 2.**
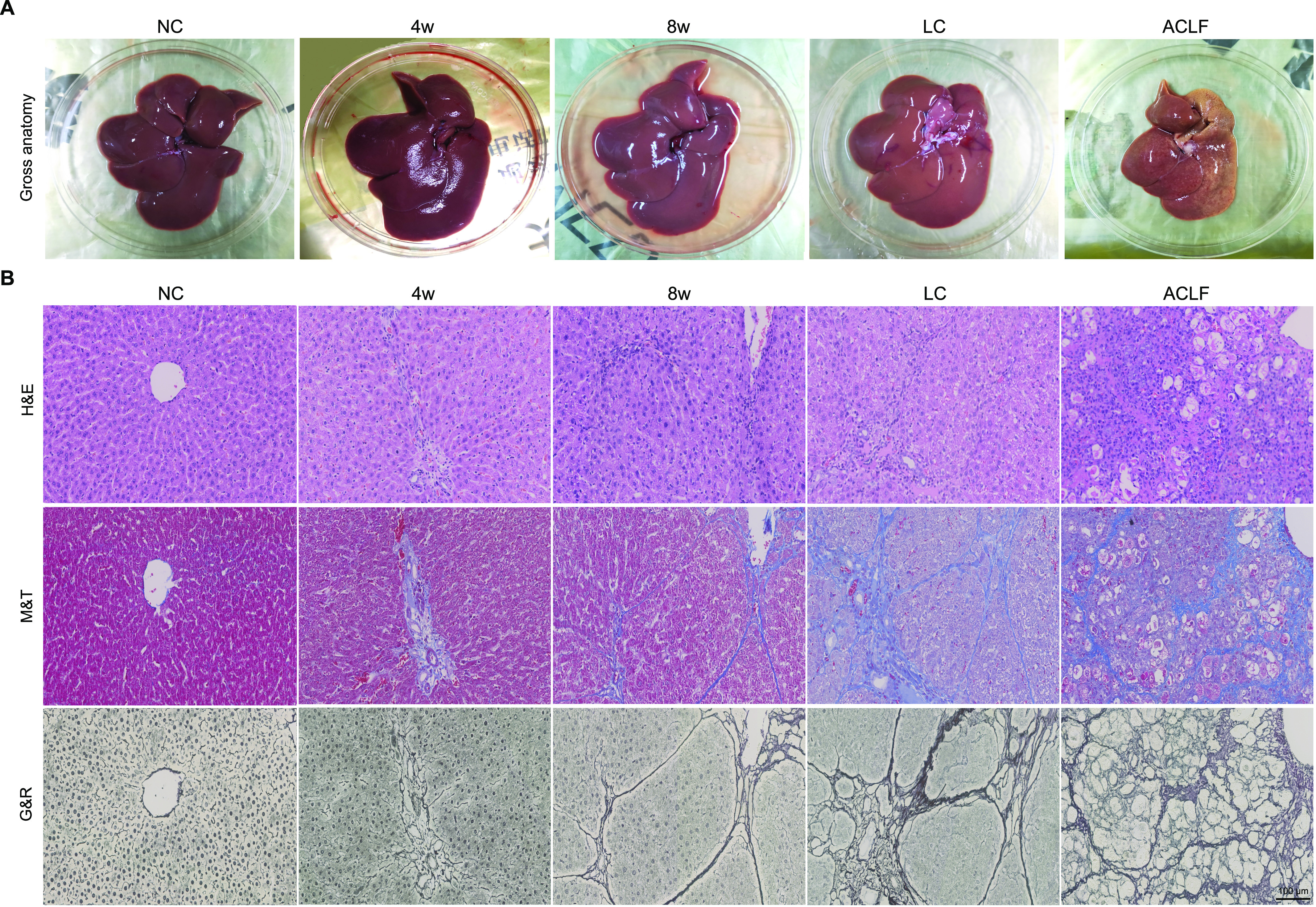
Morphological and histopathological assessment of liver samples. **(A)** Liver morphological changes in NC, LC, and ACLF rats. **(B)** Representative images of the pathological staining (H&E, M&T, and G&R) of liver sections collected from rats at different disease stages (bar = 100 μm). ACLF, acute-on-chronic liver failure; G&R Gomori’s reticulin; H&E, hematoxylin-eosin; LC, liver cirrhosis; M&T, Masson’s trichrome; NC, normal control.

Morphological inspection of rat livers revealed smooth, thin and regular liver surfaces in the NC group, whereas irregular and speckled surfaces were observed in the LC group. Moreover, visual inspection of ACLF rat livers revealed severe spotty and coarse surfaces depicting residual cirrhotic nodules, with mottled areas of bleeding and apparent extensive necrotic spots (Fig 2A). Histopathological assessment with hematoxylin–eosin (H&E) staining revealed regular liver structures in the NC group, whereas LC animals showed pronounced severe cirrhosis, as Masson’s trichrome (M&T) staining revealed classical thick-complete fibrotic septa connecting portal tracts and delimiting the classic hepatic lobules. In contrast, ACLF histological inspection showed severe distortion of the liver architecture as massive bile duct proliferation and inflammatory cell infiltration, whereas M&T staining showed entrapment of hepatocytes by fibrotic tissue, hepatocyte degeneration, ballooning, cellular necrosis, and apoptosis, and severe dissociation of the hepatic plates were present in ACLF rats (Fig 2B). In addition, Gomori’s reticulin (G&R) staining revealed the loss and collapse of the reticular fiber network that supports hepatocytes in ACLF animals, which indicates submassive necrosis and hepatocellular death. Taken together, an ACLF rat model that mimics the clinical pathogenesis has been successfully developed.

### Transcriptomic characteristics of liver tissues from the ACLF rat model

**Figure 3.**
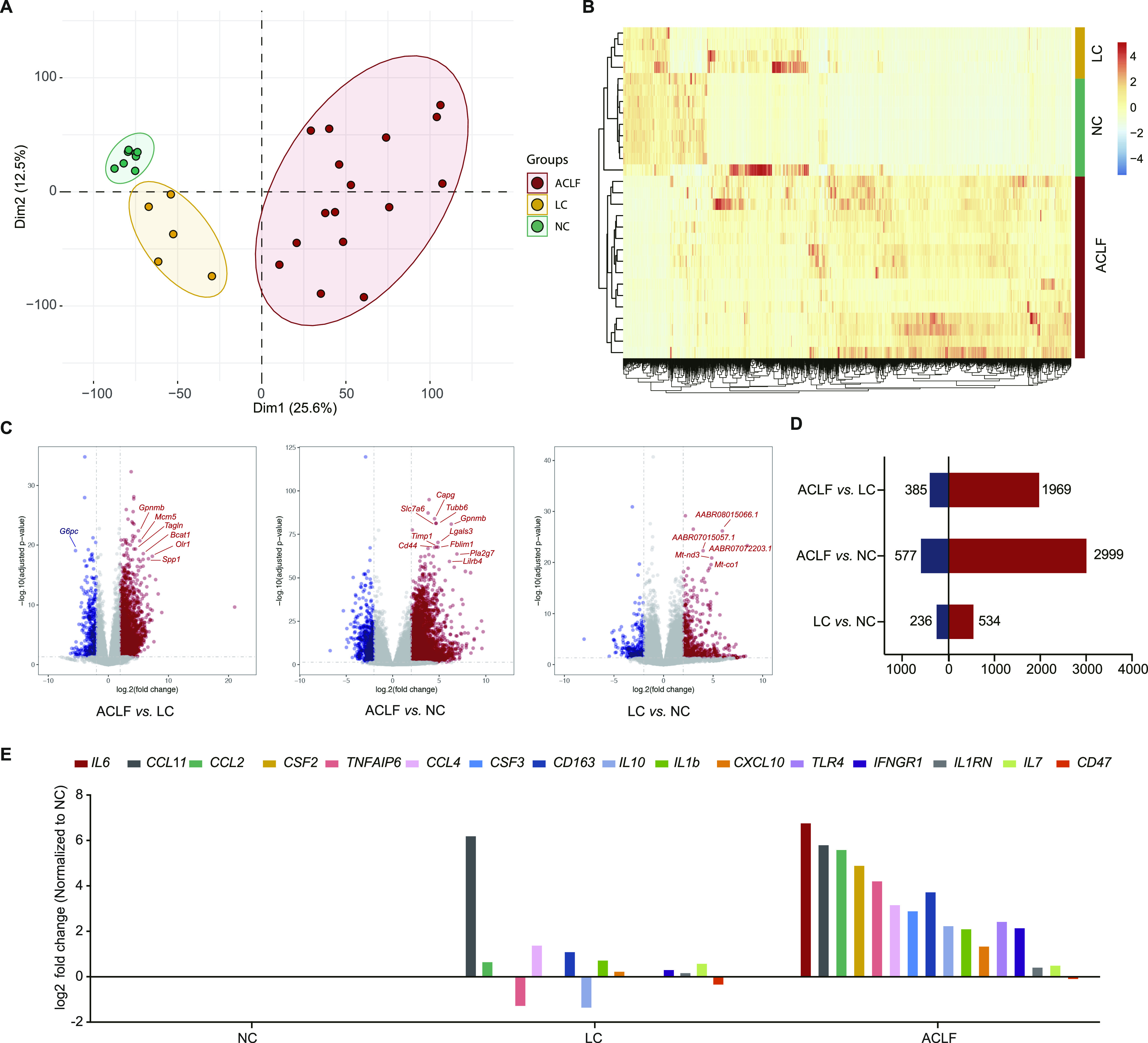
Transcriptomic characterization of the ACLF rat model. **(A)** Principal component analysis of subjects in the ACLF, LC, and NC groups. **(B)** Heat map of significant differentially expressed genes in the multigroup comparison among the three groups. **(C)** Volcano plot showing the results of pairwise differential expression analyses among the three groups. The vertical dashed lines indicate the threshold for the fourfold abundance difference. The horizontal dashed line indicates the adjusted *P*-value = 0.05 threshold. Red represents significantly up-regulated genes, whereas blue represents significantly down-regulated genes. **(D)** Number of significantly differentially expressed genes in pairwise comparisons. **(E)** The relative expression levels of genes related to the systematic inflammatory response. ACLF, acute-on-chronic liver failure; LC, liver cirrhosis; NC, normal control.

Principal component analysis (PCA) of gene expression in liver tissues showed that there were discernible differences among the ACLF, LC, and NC groups (Fig 3A), which revealed that there were considerable changes in the transcriptomic profile during the process of ACLF rat model establishment. The trajectories of the NC group, LC group, and ACLF group conformed to the ACLF rat progression. To explore the relationship between the study groups and the expression patterns of the liver tissue transcriptome, unsupervised hierarchical clustering analysis was performed based on the multigroup differentially expressed genes (DEGs). The results showed that these groups could be distinguished, and the ACLF group was the most varied group (Fig 3B). Pairwise differential expression analyses were performed using false discovery rate (FDR) filter under 0.05 with a difference of fourfold or more (Fig 3C). As indicated in Fig 3D, there was an increase in the number of significantly DEGs in the ACLF group regardless of whether NC or LC was used as the reference group (2,999 up-regulated/577 down-regulated or 1,969 up-regulated/385 down-regulated, respectively). The expression of genes related to the systemic inflammatory response was observed to describe how inflammation varied across the progression of the ACLF rat model. Typical cytokine storm induction after D-gal/LPS administration could be seen, as most inflammatory-related genes (*IL-6*, *CCl2*, *TNFAIP6*, *IL-10*, *IL-1β*, etc.) were significantly up-regulated in the ACLF stage (Fig 3E).

### Summary of functional alterations in the ACLF rat model

**Figure 4.**
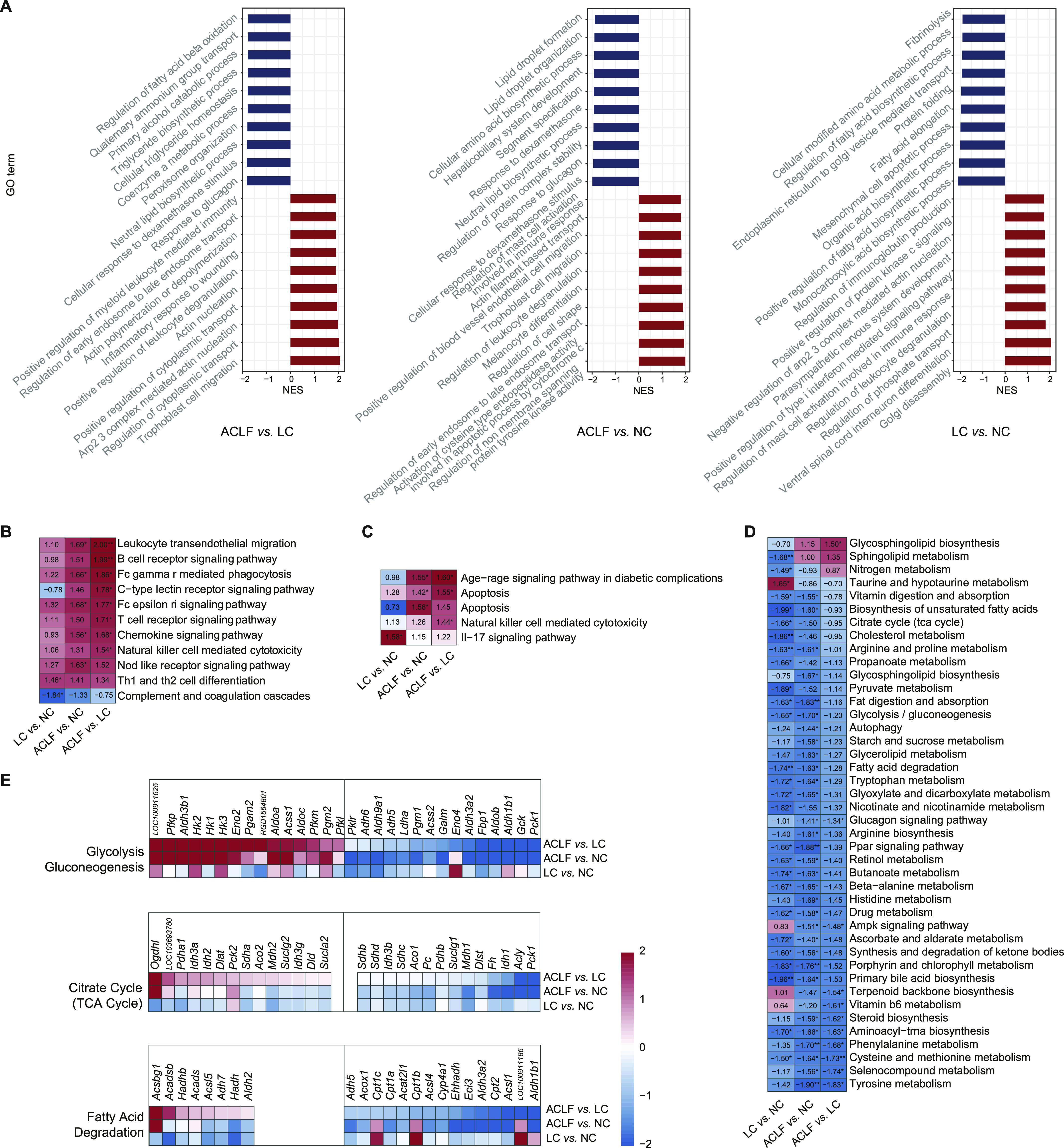
Functional alterations, the spectra of immune response and metabolic regulation during ACLF rat model development. **(A)** Identification of the top 20 significantly up-regulated and down-regulated biological processes in the pairwise comparisons among the three groups. Enrichment analysis was performed using gene set enrichment analysis (GSEA), with red and blue indicating the normalized enrichment score of each gene set. **(B, C, D)** Functional enrichment analysis of the disease groups compared with the NC groups for immune-related pathways (B), apoptosis-related pathways (C), and metabolic pathways (D) based on the Kyoto Encyclopedia of Genes and Genomes (KEGG) database. Pathways in which gene expression was increased or decreased are shown in red and blue in the heat map, respectively. The colour intensity is proportional to the normalized enrichment score calculated using GSEA. *P < 0.05, **P < 0.001. **(E)** Heat map of genes from the three main pathways. For each pathway, the top 15 up-regulated and down-regulated differentially expressed genes are shown (adjusted *P*-value < 0.05). (All differentially expressed genes are shown if the number of up-regulated or down-regulated genes was <15.) Genes with higher (red) or lower (blue) expression are proportionally displayed. ACLF, acute-on-chronic liver failure; LC, liver cirrhosis; NC, normal control.

To identify the important pathophysiological variations that occurred during ACLF rat model development, pre-ranked gene set enrichment analysis (GSEA) was performed based on the significantly correlated genes. The top 20 significantly regulated biological processes were identified across the pairwise functional analyses. As shown in Fig 4A, immune processes (e.g., regulation of myeloid leukocyte-mediated immunity and regulation of mast cell activation involved in the immune response) were significantly up-regulated in the ACLF group as compared with the LC and NC groups. Metabolic process disorders were also observed in the ACLF group (e.g., regulation of fatty acid β oxidation, glycerolipid metabolism, and cellular amino acid biosynthetic process) compared with the LC and NC groups. These observations showed the prominent dysregulation of immune processes and metabolic processes during the development of the ACLF rat model, which were closely related to the known clinical manifestations of ACLF. Based on these findings, our ACLF rat model could depict the clinical pathogenesis of ACLF.

### Immune-metabolism disorder in the ACLF rat model

After observing the significant variation in immune processes and metabolic processes, we aimed to further explore immune and metabolic changes during the development of the ACLF rat model based on correlated pathways from the Kyoto Encyclopedia of Genes and Genomes (KEGG) database. As shown in Fig 4B, the enrichment analysis of immune-related pathways revealed that the immune response was significantly up-regulated in ACLF throughout our ACLF rat model development, and B-cell receptor signaling pathway, leukocyte transendothelial migration, and chemokine signaling pathways were the most significantly changed (*P* < 0.01), whereas apoptosis considered as principal pathway (Fig 4C). In addition, Fig 4D showed the dysregulation of metabolic-related processes such as fatty acid degradation, citrate cycle (TCA cycle), and primary bile acid biosynthesis, progressively increased from the LC to ACLF group (*P* < 0.01). The expression levels of genes encoding key products involved in the target pathways were compared in the three groups, and robust changes were observed throughout ACLF rat model development (Fig 4E). Overall, the ACLF group displayed a significant reduction in the expression of metabolic genes compared with that in the LC and NC groups, especially for genes related to glycolysis/gluconeogenesis, the TCA cycle and fatty acid degradation.

### Immune and hepatic cell microenvironment patterns during ACLF rat model development

**Figure 5.**
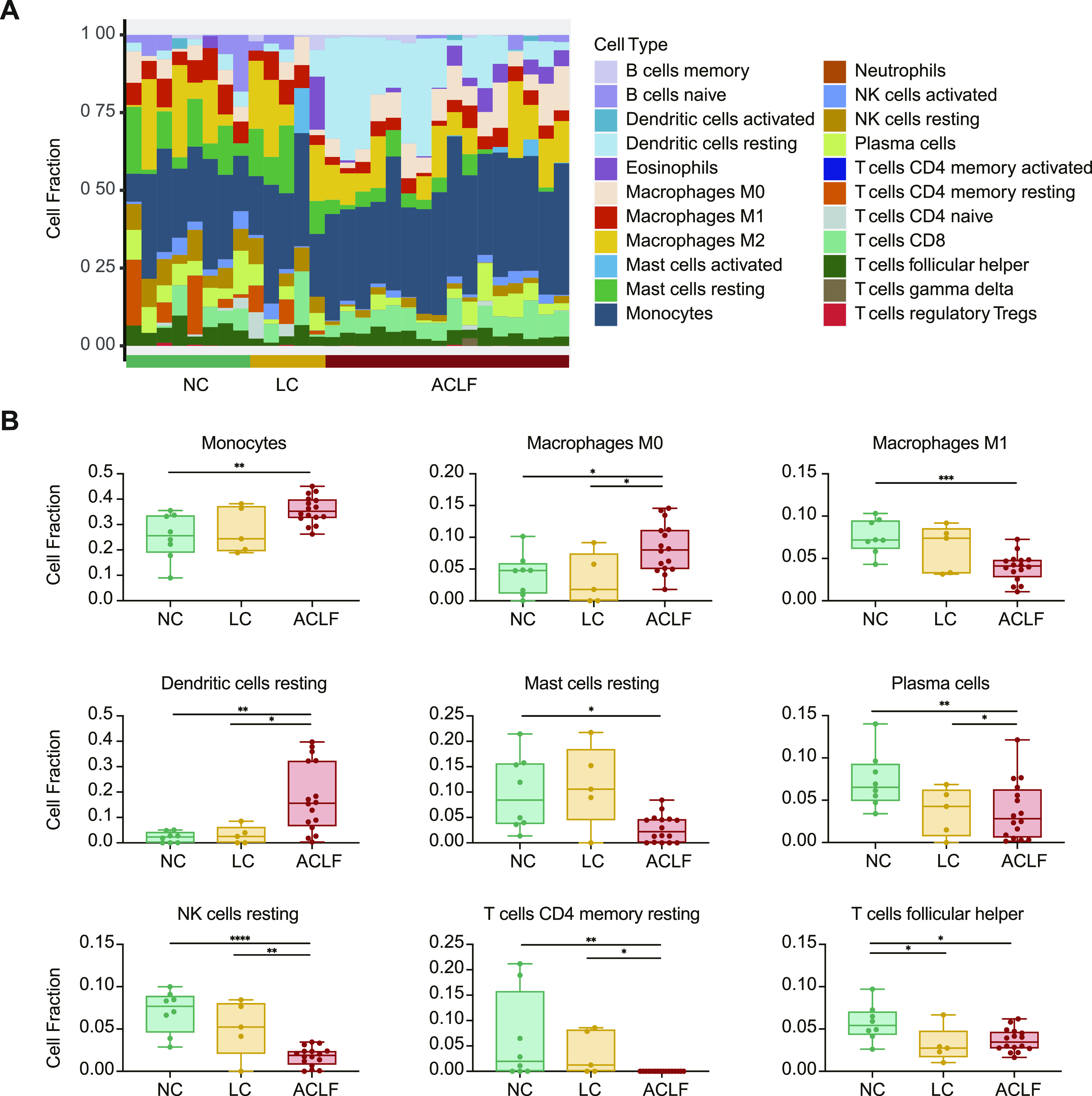
The change of immune microenvironment with ACLF development. **(A)** CIBERSORT cellular composition analysis revealed relative fractions of different immune cell types varied across ACLF development. **(B)** The relative fraction changes of immune cell types associated with ACLF rat progression. *P < 0.05, **P < 0.01, ***P < 0.001. ACLF, acute-on-chronic liver failure; LC, liver cirrhosis; NC, normal control; NK, natural killer.

As highlighted in Fig 5A, the analysis of the immune cell fractions during ACLF development showed significant variation in immune cellular composition between NC, LC and ACLF groups. The fractions of monocyte, macrophages M0, and resting dendritic cells, representing the innate immune response, were significantly higher in the ACLF group compared with LC and NC groups, whereas resting mast cells, plasma cells, resting NK cells, CD4 memory resting T cells, and follicular helper T cells were significantly lower in the ACLF group (Fig 5B). Eosinophils and neutrophils were not obviously altered between the NC, LC, and ACLF groups.

**Figure S1.**
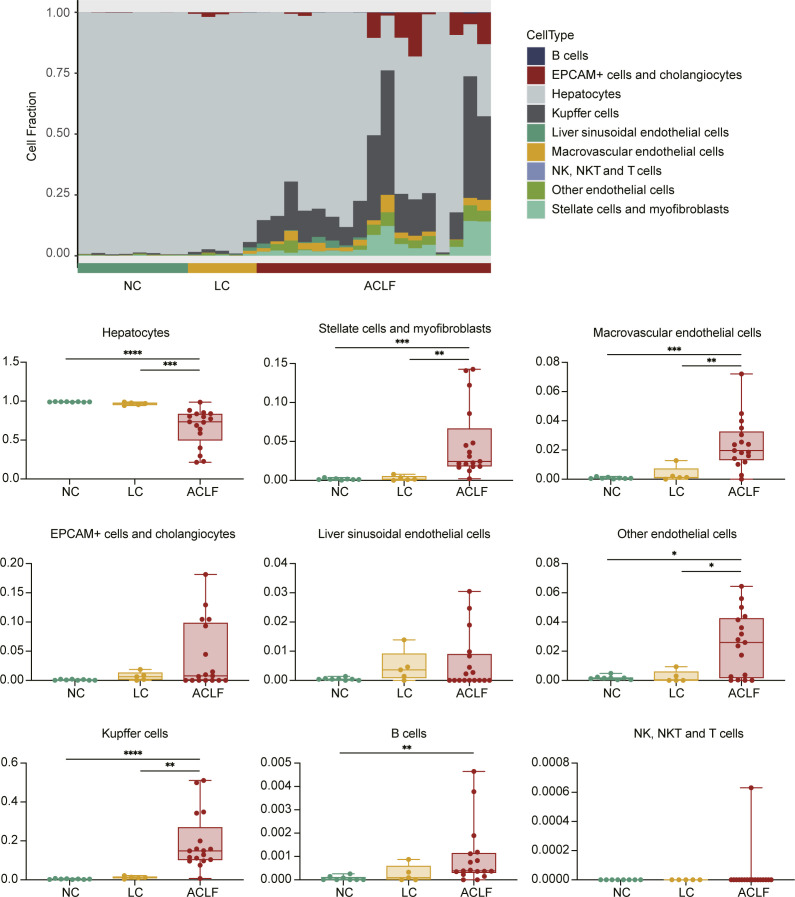
Liver cells deconvolution across ACLF development. The relative fraction changes of hepatic cell types associated with ACLF rat progression. *P < 0.05, **P < 0.01, ***P < 0.001, ****P < 0.0001. ACLF, acute-on-chronic liver failure; LC, liver cirrhosis; NC, normal control.

Moreover, liver cell deconvolution analysis showed significant lower fraction of hepatocytes in the ACLF group compared with the LC and NC groups. Interestingly, fractions of stellate cells, myofibroblasts, and Kupffer cells were significantly higher in the ACLF group (Fig S1).

### Functional signatures associated with ACLF development

**Figure 6.**
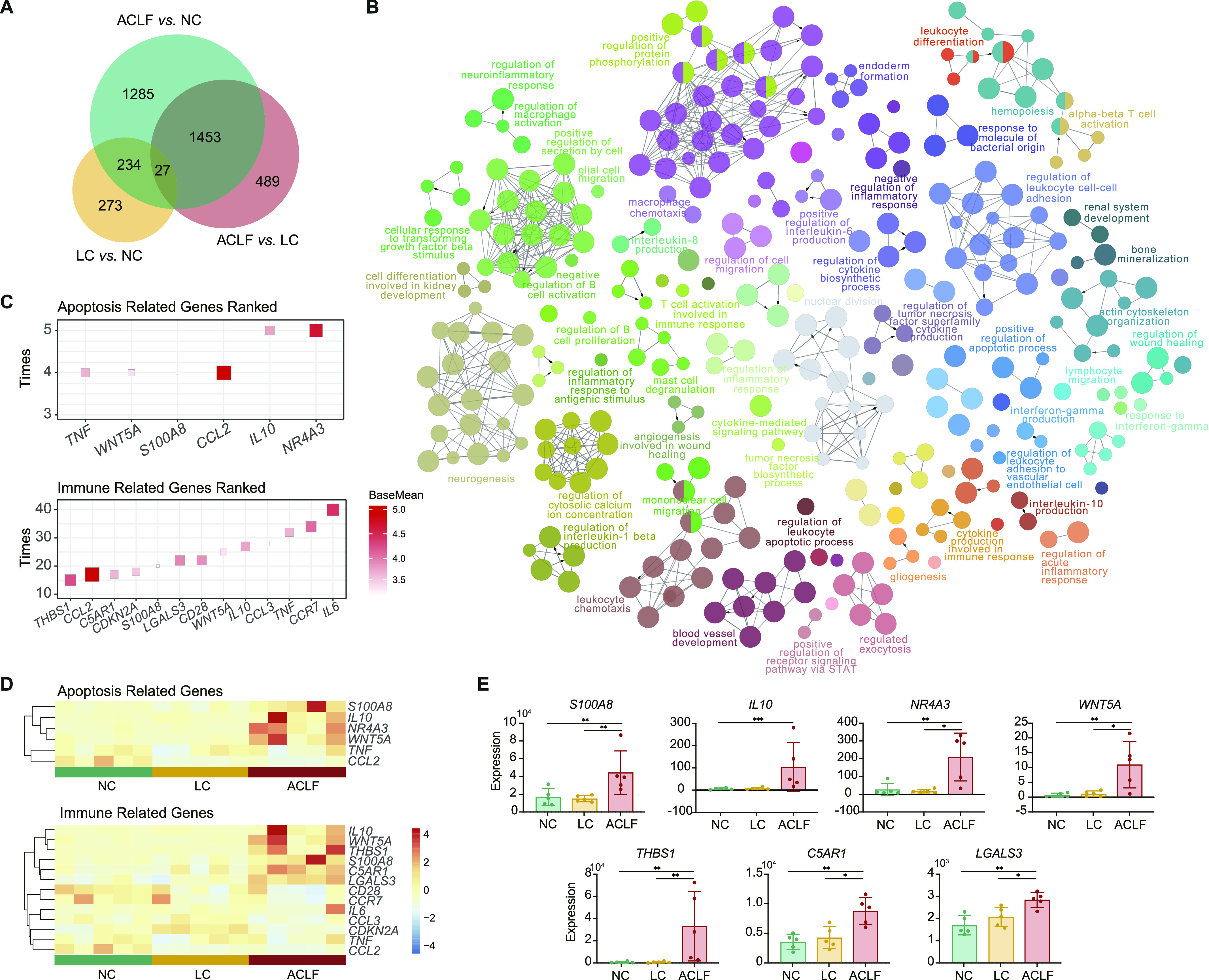
Functional signatures associated with ACLF development. **(A)** Venn diagram of the differentially expressed genes analyzed in pairwise comparisons among the ACLF, LC, and NC groups. **(B)** Network of the biological processes identified in a functional enrichment analysis of 1,453 overlapping differentially expressed genes. **(C)** The frequencies of the top genes appearing in the apoptosis-related (top) and immune-related (bottom) biological processes. **(D)** Heat map based on the RNA-Seq data of ACLF patients, LC patients and healthy subjects (n = 5 each) for the functional genes. **(E)** The expression of seven genes (*THBS1*, *IL-10*, *NR4A3*, *S100A8*, *WNT5A*, *C5AR1*, and *LGALS3*) in human samples, which were in accordance with the expression trend of ACLF rats. * adjusted *P*-value < 0.05, ** adjusted *P*-value < 0.01, *** adjusted *P*-value < 0.001. ACLF, acute-on-chronic liver failure; C5AR1, complement component 5a receptor 1; IL-10, interleukin-10; LC, liver cirrhosis; LGALS3, galectin-3; NC, normal control; NR4A3, nuclear receptor 4A3; S100A8, S100 calcium-binding protein A8; THBS1, thrombospondin-1.

A total of 1,453 identified genes were differentially expressed both in the comparisons of ACLF versus LC and ACLF versus NC, which were related to ACLF pathophysiology (Fig 6A). The functional analysis showed that these genes were mostly involved in immune-related and apoptosis-related biological processes, such as “T-cell activation involved in immune response,” “interleukin-8 production,” “regulation of acute inflammatory response,” and “positive regulation of apoptotic process” (Fig 6B). To identify the key molecules that were associated with ACLF, the frequencies of appearance of genes in apoptosis-related and immune-related biological processes were counted (Fig 6C). The potential key molecules related to apoptosis and immune were validated in the RNA-Seq data of patients with ACLF (Fig 6D). The results showed that seven genes with increased expression during the progression from NC, LC to ACLF, which were in consistent with the gene expression detected in the ACLF rats (Fig 6E). These results indicated the potential clinical use of these seven genes as diagnostic and prognostic biomarkers for ACLF onset and prognosis.

### Validation of the three potential biomarkers of ACLF pathogenesis

**Figure 7.**
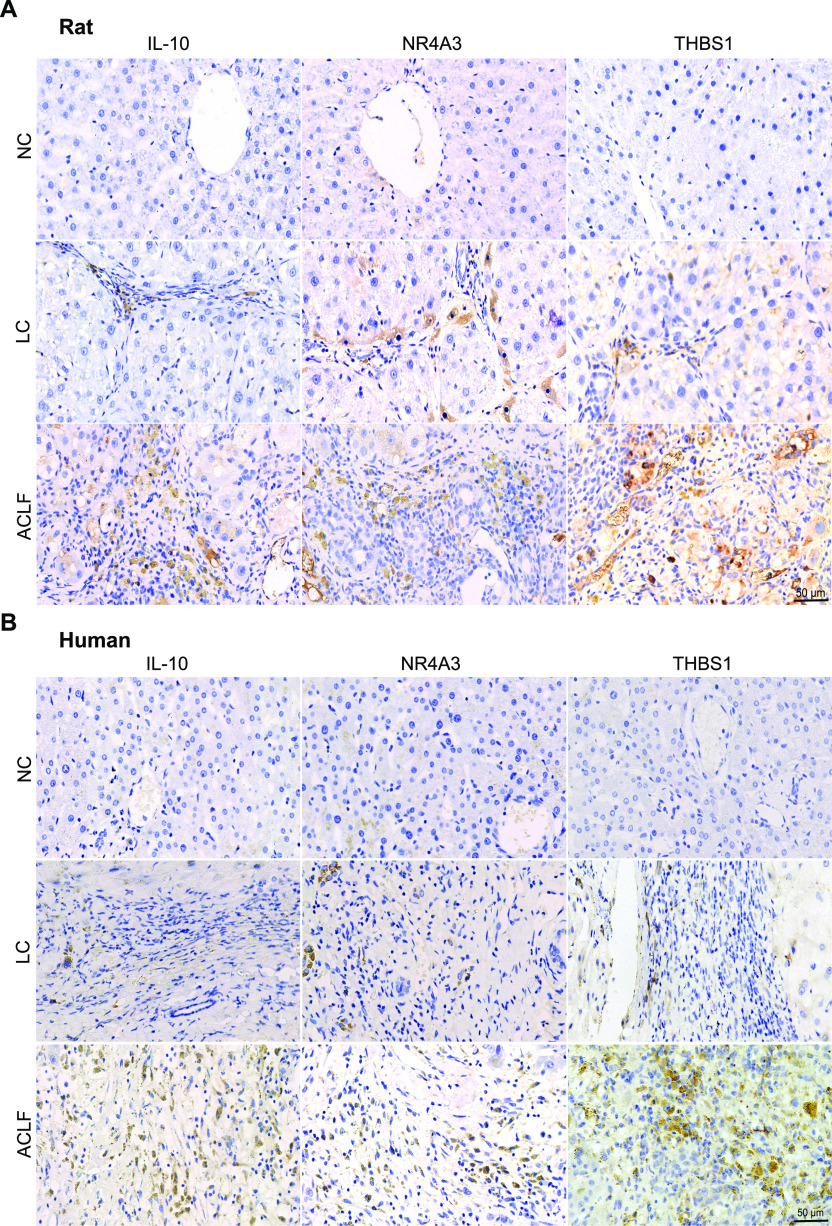
**Validation of the biomarkers related to immune-metabolism disorder. (A, B)** Representative immunohistochemistry (IHC) staining of the three potential immune-metabolism biomarkers (THBS1, IL-10, and NR4A3) of ACLF development in rats (A) and humans (B) liver tissues (bar = 50 μm). ACLF, acute-on-chronic liver failure; IL-10, interleukin-10; LC, liver cirrhosis; NC, normal control; NR4A3, nuclear receptor 4A3; THBS1, thrombospondin-1.

Among the seven DEGs related to immune-metabolism disorder as the core molecular mechanism underlying the ACLF pathogenesis, IL-10, NR4A3, and THBS1 proteins were externally validated by immunohistochemistry (IHC) in ACLF rats (Fig 7A) and patients liver biopsies (Fig 7B), in which their expressions were increased from NC and LC to ACLF, confirming their specificity patterns in ACLF disease progression.

## Discussion

For better understanding of ACLF pathogenesis, preventing the development of the life-threatening complications and improving the clinical intervention strategies, it is crucial to establish an animal model that entirely mimics ACLF disease spectrum. The detailed transcriptomic assessment of such model could efficiently reveal the ACLF disease molecular mechanism.

In this study, we developed ACLF in cirrhotic rats via D-gal/LPS acute insult. LC stage was induced by PS administration, as the repetition of toxic insults to the liver is a classic way to induce liver fibrosis/cirrhosis in experimental animals (Weber & Wasmuth, 2010), as well as diet-based, surgical, or genetic modification and infectious-based models (Liu et al, 2013; Yanguas et al, 2016), but the application of these approaches is questionable because of the very long time required to reach stable cirrhosis, abrupt animal death during LC development, and the very short survival period after ACLF induction (Xiang et al, 2020). Immune-mediated CLD animal models are feasible through administration of heterologous serum that could generate LC with minimal hepatocellular damage (Tsukamoto et al, 1990; Baba et al, 2005). The application of PS has been adopted to induce LC, which is superior to human serum albumin in terms of model stability, consistency, and the absence of high mortality, as no animal died during the cirrhotic stage of ACLF development in our study, whereas Li et al (2017) previously reported that in a human serum albumin–induced ACLF rat model, almost 23% of rats died during the fibrotic stage of model development (Li et al, 2013). Wang et al (2012, 2014, 2016) and Li et al (2017) previously reported similar ACLF rat model applying PS and D-gal/LPS, but their findings lack the typical clinical features of LC, which might be due to the PS dosage duration and/or the selected experimental animal species.

In the present work, we selected Sprague–Dawley (SD) rats over Wistar and other strains because of their relative sensitivity to PS-induced LC, as previously described by Baba et al (2004), as signs of fibrosis appeared as early as 4 wk and continued to progress with time till consistent LC. Acute insult of cirrhotic rats promoted ACLF generation, as D-gal/LPS initiated a typical cytokine storm characterized by massive secretion of proinflammatory mediators and soluble cytokines, which trigger hepatic inflammation and subsequent tissue injury (Hamesch et al, 2015; Martin et al, 2015). ACLF rats showed typical characteristics manifested by liver dysfunction, significant cirrhotic nodules surrounded by fibrotic septa, massive necrosis, and cellular apoptosis accompanied by elevation in the parameters reflecting renal dysfunction. In summary, compared with mild hepatic injury and immense animal death in a short time in the previous PS-based ACLF model, our rat model showed stable LC and massive hepatic failure in ACLF stages up to 72 h after acute insult.

Transcriptomic characterization of liver tissues from ACLF rats collected at different disease stages revealed immune-metabolism disorder as the core mechanism underlying ACLF development and prognosis. The dramatic alteration of innate immune response typically depends on the immune cells stimulation and associated with repressive adaptive immune response. Previously, we and others reported that signatures related to innate immune response (interferon-related, monocyte-related, and neutrophil-related) were significantly expressed while those related to adaptive immune response (T-cell–related, B-cell–related, and NK-cell–related) were exhausted in ACLF patients (Clària et al, 2016; Weiss et al, 2020; Li et al, 2021). In the present study, further assessment of immune cell distribution among ACLF disease stages confirmed the fundamental accumulation of monocyte, macrophages M0, and resting dendritic cellular fractions in the ACLF group, which could trigger the release of soluble inflammatory components and typical cytokine storm, whereas loss of the functional hepatocyte as a result of massive necrosis and apoptosis might trigger the hepatic/extrahepatic organ failure. Meanwhile, the deteriorated metabolic processes, manifested by severe disruption of glycerolipid metabolism and primary bile acid biosynthesis, might indicate the massive loss of functional hepatocytes. Previous reports correlated the ACLF prognosis with glycolysis metabolic dysregulation and inhibition of mitochondrial energy production (Moreau et al, 2020; Li et al, 2021). A recent study assessed the mitochondrial morphology and central metabolic pathways with emphasis on the tricarboxylic acid (TCA) cycle, and the findings leverage the appreciation for the role of mitochondrial dysfunction in the advanced liver diseases (Zhang et al, 2021). Taken together, these findings revealed immune-metabolism disorder as the core mechanism underlying ACLF development and prognosis; in this context our model resembles the human clinical ACLF manifestations, regardless of the ACLF disease etiology and/or precipitating event.

Identification of reliable biomarkers related to immune-metabolism disorder is a critical predictor of ACLF severity prognosis and/or facilitates timely intensive clinical interventions (Mookerjee, 2016). Our discovered three biomarkers from ACLF rats (*IL-10*, *NR4A3*, and *THBS1*) were externally validated in patients’ transcriptomics, rats and humans’ liver samples using IHC. Recently, *IL-10* has been reported to be valid prognostic biomarker of ACLF (Wang et al, 2014, 2016). *NR4A3*, a member of the NR4A nuclear receptor family, has shown to induce apoptosis in liver tissues (Herring et al, 2019). *THBS1* was certified in regulating immune activation, enhancing inflammatory response, and accelerating fibrosis (Lopez-Dee et al, 2011). *S100A8* strongly correlated with activation of proinflammatory mediators and indices of ACLF disease severity, extrahepatic organ failure, and outcome (Singanayagam et al, 2018). *WNT5A* overexpression has been positively correlated with ACLF prognosis (Ji et al, 2015). *C5AR1* contributed to induction of cytokine-mediated inflammation in the liver (McCullough et al, 2016). *LGALS3* levels extensively reflect the degree of liver fibrosis (Gudowska et al, 2015). The comprehensive molecular mechanisms of these biomarkers need to be thoroughly investigated. Application of integrated investigational approaches with suitable HBV-ACLF animal model using flow cytometry, proteomics, metabolomics and bioenergetic data, with Seahorse system, should be performed in the near future to decipher disease pathogenesis.

Overall, we developed stable ACLF rat model that mimics the clinical pathogenesis which indicates immune-metabolism disorder is indispensable in ACLF pathogenesis. This finding will be of great interest for clinicians in providing new targets for improving intervention strategies and may lead to beneficial clinical applications in the future.

## Materials and Methods

### Drugs and chemicals

PS (Cat. no. 26250084) was obtained from Gibco. D-gal (Lot No SLBM3382V, analytical standard ≥99%) and LPS (Lot No BCBR3574V, derived from *Escherichia coli* 0128:B12 serotype, source strain is CDC 2440-69) were purchased from Sigma-Aldrich.

### Reagents

Serum biomarker detection reagents were equipment-related reagents. Pathological section-related reagents were provided by the Department of Pathology, The First Affiliated Hospital, Zhejiang University School of Medicine. Additional solvents and reagents of the highest standard analytical grade were commercially available.

### Experimental animals

Male, SD, specific pathogen-free grade rats weighing 60–80 g (age 4 wk) were provided by Zhejiang Academy of Medical Sciences Animal Center (animal license number SCXK 2019-0002). All animal experiments comply with the ARRIVE guidelines and the experimental procedures were conducted in accordance with the National Institute of Health (NIH) guidelines for the care and use of laboratory animals, and the study was ethically approved by the Ethics Committee of the First Affiliated Hospital, Zhejiang University School of Medicine (approval No. 008-2018). Rats were maintained in a specific pathogen-free facility with controlled environmental conditions (22°C ± 1°C, 55% ± 5% relative humidity, 12 h light–dark cycle) with free access to standard rodent chow and water ad libitum.

### Experimental design and model establishment

After 1 wk of acclimatization, the rats were randomly allocated into two groups (NC group: n = 35; ACLF group: n = 65). In the ACLF group, the rats were intraperitoneally administered PS at a 2 ml/kg dose (Huang et al, 2019) twice a week for 12 consecutive weeks to generate LC. At 4, 8, and 12 wk post PS administration, eight rats were randomly selected and euthanized to assess the development and progression of liver fibrosis/cirrhosis. After 12 wk, rats with LC were intraperitoneally injected with the D-gal/LPS combination (800 mg/kg D-gal and 100 μg/kg LPS) to induce acute liver failure on the basis of chronic LC (Liu et al, 2009; Wang et al, 2012), whereas rats in the NC group were administered normal saline. Then, the animals were separated into an experimental group and a survival group. 72 h after D-gal/LPS administration, the rats were euthanized under anesthesia, and blood samples were collected for serum isolation. Liver samples were removed, snap-frozen in liquid nitrogen, and then stored for additional experiments.

### Patient selection

To further explore and confirm the potential research value of ACLF rat model, patients with ACLF based on cirrhosis (n = 5) were enrolled in this study. The diagnostic criteria for the patients with ACLF were based on the CANONIC study (Moreau et al, 2013). As controls, patients with LC (defined as patients with stable compensated cirrhosis, n = 5) and healthy individuals (n = 5) were also participated in the study. The relevant clinical characteristics of patients and healthy controls were shown in Table S1. PBMCs from the 15 subjects were subjected to transcriptomic sequencing.

Table S1 Clinical characteristics of patients in sequencing group.

### Serum biomarker determination

TP, ALB, ALT, AST, TBA, TBil, γGT, Cr, and BUN levels were measured with autoanalyzer (Roche Cobas 8000) in accordance with standard spectrophotometric methods to assess liver function.

### Assessment of serum fibrotic marker

HA has been found to positively correlate with the histological stages of liver fibrosis in chronic liver diseases and has shown very good diagnostic accuracy for the noninvasive assessment of liver fibrosis/cirrhosis (Gudowska et al, 2017). Serum HA levels were assessed by using an automatic clinical chemistry analyzer (AU5800, Beckman).

### Histological examination of liver tissues

Subsequent to their isolation, livers were promptly subjected to histopathological analyses, as they were fixed in 10% paraformaldehyde solution and embedded in paraffin wax. To observe the pathological features and ACLF progression, liver tissues from different time points were subjected to H&E, M&T, and G&R staining. Each liver tissue section was heat-fixed at 60°C for 1 h and then stained with H&E, as previously described (Li et al, 2012). M&T staining was performed according to recommended protocols (Maixin Biotech and Solarbio Life Science), as previously illustrated (Yuan et al, 2019). G&R staining was conducted in accordance with current staining practice, as previously described (Kvasnicka et al, 2016). Investigators were blind during findings assessment.

### Total RNA extraction and mRNA-seq

Freshly isolated, snap-frozen liver tissue samples were homogenized in TRIzol reagent (Invitrogen Life Technologies), and total RNA was extracted following the manufacturer’s instructions. A sequencing library was then prepared according to the manufacturer’s instructions (TruSeq RNA LT Sample Prep Kit v2, Illumina), including the mRNA purification and fragmentation, first-strand cDNA synthesis, second-strand cDNA synthesis, end repair via adenylation of the 3′ ends, adapter ligation, and DNA fragment amplification steps. The pooled library consisted of sequences with lengths of ∼250 nucleotides. The library was sequenced using the HiSeq 2500 sequencing system (Illumina). The average number of sequencing reads was ∼71.9 million per RNA sample.

Adaptors and low-quality reads were removed using Trimmomatic v0.36 with the default parameters. Paired-end transcriptome sequencing reads from each sample were aligned with a rat reference genome (Rnor 6.0) using HISAT v2.0.5 with the default parameters. HTSeq v0.6.18 was used to compute the raw read counts for each gene. DESeq2 v1.14.1 was used to identify DEGs between the two groups. Significance was defined as an “adjusted *P*-value < 0.05” in detecting transcript changes, unless indicated otherwise. The FDR calculated with the Benjamini–Hochberg procedure (as implemented in the R function *P*.adjust) was used to control type I error in multiple tests.

### PCA and unsupervised hierarchical clustering analysis

To reduce the number of features, PCA was performed based on the normalized read count (R, “FactoMineR” package). To estimate the similarity among different samples, unsupervised hierarchical clustering analysis was implemented based on the significant DEGs. The R function “pheatmap” was used to plot the gene expression heat map and perform hierarchical clustering of DEGs obtained. Pearson’s correlation coefficient was used as a distance measure in clustering. A complete linkage method was used for all the tests.

### Functional interpretation of variations in expression

To illustrate the alterations in biological processes throughout our ACLF rat model development, functional enrichment analysis was performed using GSEA based on the differential expression between the two groups. Gene Ontology was used to depict the changes in biological processes, and the KEGG database was used in the study to further investigate immune and metabolic alterations across model development stages. The significance criterion used for the GSEA was a *P*-value < 0.05. The NES was calculated by GSEA to compare the degree of enrichment across gene sets.

### Calculation of immune and hepatocyte cell fractions

To characterize the variation in immune composition across ACLF development, CIBERSORT was adopted to infer the relative fractions of 22 different immune cells types in the samples. It is a deconvolution algorithm based on gene expression profiles, which quantifies the immune cell relative fractions using per cellular signatures. In the meantime, liver cell fractions were quantified to assess the hepatocellular alteration during ACLF progression.

### Functional enrichment analysis

The annotation enrichment analysis of DEGs was performed using ClueGO in Cytoscape. Bonferroni step-down method was used to adjust the FDR, and Gene Ontology terms with adjusted *P*-value < 0.05 were considered significantly enriched.

### Assessment of the potential biomarker expressions

The expression of potential proteins encoded by the target genes were confirmed by IHC in liver tissues derived from ACLF rats and human biopsies. The indicated antibodies corresponding to these biomarkers were listed in Table S2. The IHC images were analyzed with 3DHISTECH 2.2 CaseViewer software.

Table S2 Antibodies for immunohistochemistry (IHC).

## Data Availability

All data associated with this study are present in the paper or the supplementary materials. Sequencing reads are available in the National Center for Biotechnology Information (NCBI) Sequence Read Archive database (https://www.ncbi.nlm.nih.gov/sra) with the accession numbers: PRJNA713912, PRJNA548207.

### Statistical analysis

Experimental data, presented as the mean ± SD, were statistically analyzed using GraphPad Prism 8 (GraphPad Software, Inc.). Significance was determined using ANOVA and a probability level of a *P*-value of ≤0.05 was considered statistically significant.

## Supplementary Material

Reviewer comments
