# Supplementary material for: Transcriptomics reveals immune-metabolism disorder in acute-on-chronic liver failure in rats
Source: Life Sci Alliance. 2021 Dec 1;5(3):e202101189. doi: 10.26508/lsa.202101189 (PMC8645333; doi:10.26508/lsa.202101189)
Supplement: Supplementary file 1 [file LSA-2021-01189_TableS1.docx]

**Supplementary Table S1.** Clinical characteristics of patients in sequencing group.

| **Characteristics** | **ACLF (n=5)** | **LC (n=5)** | **NC (n=5)** | ***P* value** |
| --- | --- | --- | --- | --- |
| Age (yrs.) | 44.8±8.0 | 47.8±4.4 | 45.8±12.5 | 0.833 |
| Male (No.) | 100% (5) | 100% (5) | 100% (5) | 0.475 |
| **HBV-DNA level (IU/ml)** |  |  |  | 0.004 |
| ≤ 2×10^2^ | 20.0% (1) | 0 | 0 |  |
| 2×10^2^ - 2×10^6^ | 40.0% (2) | 0 | 0 |  |
| > 2×10^6^ | 40.0% (2) | 100.0% (5) | 100.0% (5) |  |
| **Laboratory data** |  |  |  |  |
| Alanine aminotransferase (U/L) | 193.0 [160.0, 368.0] | 20.2±11.9 | 32.8±21.7 | 0.002 |
| Aspartate aminotransferase (U/L) | 105.0 [80.0, 171.0] | 25.2±5.7 | 26.8±7.6 | 0.001 |
| Albumin (g/L) | 33.9±2.9 | 46.3±3.7 | 47.7 [47.1, 48.1] | <0.001 |
| Total bilirubin (μmol/l) | 448.6±129.2 | 17.0±5.2 | 11.0±2.1 | <0.001 |
| Alkaline phosphatase (U/L) | 153.4±53.8 | 94.2±14.6 | 63.4±30.0 | 0.004 |
| γ-glutamyl transpeptidase (U/L) | 90.0±41.0 | 35.6±18.0 | 38.4±26.6 | <0.001 |
| Creatinine (μmol/L) | 58.4±10.2 | 72.5±13.0 | 85.4±4.9 | 0.002 |
| Sodium (mmol/L) | 134.2±4.3 | 142.0±0.8 | 142.6±2.1 | <0.001 |
| White blood cell count (10^9^/L) | 6.1 [5.8, 8.3] | 4.5±1.1 | 6.1±0.8 | 0.100 |
| Hemoglobin (g/L) | 136.2±8.0 | 156.4±6.4 | 154.5±10.6 | 0.012 |
| Hematocrit (%) | 38.6±2.9 | 46.1±2.2 | 46.2±3.0 | 0.002 |
| Platelet count (10^9^/L) | 65.6±21.8 | 110.6±30.8 | 261.5±0.7 | 0.002 |
| INR | 2.6±0.5 | NA | NA | 0.143 |
| Alpha fetoprotein (μg/L) | 154.7±88.8 | 2.2±0.8 | 2.4±0.8 | <0.001 |
| **Organ failure (No.)** |  |  |  |  |
| Liver | 100.0% (5) | NA | NA |  |
| Coagulation | 80.0% (3) | NA | NA |  |
| Kidney | 0 | NA | NA |  |
| Cerebral | 0 | NA | NA |  |
| Lung | 0 | NA | NA |  |
| Circulation | 0 | NA | NA |  |
| **Severity score** |  |  |  |  |
| MELD | 25.5±1.4 | NA | NA |  |
| MELD-Na | 27.7±1.5 | NA | NA |  |
| CLIF-C ACLFs | 44.3±1.8 | NA | NA |  |
| **Transplant-free mortality rate** |  |  |  |  |
| 28-day | 40% (2) | NA | NA | 0.007 |
| 90-day | 60% (3) | NA | NA | <0.001 |

NOTE. Data are expressed as the mean ± standard deviation (SD) or percentages (number of patients).

ACLF, acute-on-chronic liver failure; LC, liver cirrhosis; NC, normal control.
