# Supplementary material for: Transcriptomics reveals immune-metabolism disorder in acute-on-chronic liver failure in rats
Source: Life Sci Alliance. 2021 Dec 1;5(3):e202101189. doi: 10.26508/lsa.202101189 (PMC8645333; doi:10.26508/lsa.202101189)
Supplement: Supplementary file 2 [file LSA-2021-01189_TableS2.docx]

**Supplementary Table S2.** Antibodies for Immunohistochemistry (IHC)

| **Primary antibodies** | | |  |
| --- | --- | --- | --- |
| Antibody | Catalog number | Company | Dilution |
| *THBS1* | ab1823 | Abcam, Cambridge, UK | 1:2000 |
| *IL-10* | orb319439 | Biorbyt, Cambridge, UK | 1:1000 |
| *NR43A* | orb479105 | Biorbyt, Cambridge, UK | 1:500 |
